# A Least Square Method Based Model for Identifying Protein Complexes in Protein-Protein Interaction Network

**DOI:** 10.1155/2014/720960

**Published:** 2014-10-23

**Authors:** Qiguo Dai, Maozu Guo, Yingjie Guo, Xiaoyan Liu, Yang Liu, Zhixia Teng

**Affiliations:** ^1^School of Computer Science and Technology, Harbin Institute of Technology, P.O. Box 319, 92 Xidazhi Street, Harbin 150001, China; ^2^School of Information and Computer Engineering, Northeast Forestry University, Harbin 150040, China

## Abstract

Protein complex formed by a group of physical interacting proteins plays a crucial role in cell activities. Great effort has been made to computationally identify protein complexes from protein-protein interaction (PPI) network. However, the accuracy of the prediction is still far from being satisfactory, because the topological structures of protein complexes in the PPI network are too complicated. This paper proposes a novel optimization framework to detect complexes from PPI network, named PLSMC. The method is on the basis of the fact that if two proteins are in a common complex, they are likely to be interacting. PLSMC employs this relation to determine complexes by a penalized least squares method. PLSMC is applied to several public yeast PPI networks, and compared with several state-of-the-art methods. The results indicate that PLSMC outperforms other methods. In particular, complexes predicted by PLSMC can match known complexes with a higher accuracy than other methods. Furthermore, the predicted complexes have high functional homogeneity.

## 1. Introduction

Proteins do not function in isolation but interact together to form complexes. Protein complex plays an important role in cellular activities, such as signal transduction, cell cycle, DNA transcription, and DNA repair [1–3]. Identifying protein complexes is crucial for understanding molecular mechanism in cellular activities. It is important to develop computational methods for identifying complexes [1]. Recent developments in high-throughput technologies have produced large amount of high-quality protein-protein interaction (PPI) data that can be represented as a PPI network, an undirected graph, in which nodes denote that proteins and edges are interactions between pairs of proteins. Graph clustering techniques are used to identify protein complexes by finding dense regions in a PPI network [4]. Since proteins may belong to several complexes, most of previous methods detect overlapping clusters [1, 4–6].

Many methods [7–9] detect complexes from PPI network by finding cliques, in which all nodes connect to each other. CFinder is one of the most popular clique-based methods, which searches adjacent cliques in the network [8, 10, 11]. OCG [12] takes the cliques as initial classes for hierarchy fusion to detect overlapping clusters in PPI networks. Another kind of methods detects complexes by expanding a set of seed proteins or clusters. MCODE [13] chooses the proteins with high weights as seeds and expands these seeds by including their neighboring proteins with weights higher than a threshold. ClusterONE [14], the latest and powerful seed-expansion method, starts from a set of seed complexes and expands them by maximizing the cohesiveness function. The expanding method depends on the density-based definition of the complexes. Random walking techniques have been also used to detect complexes. Markov clustering (MCL) algorithm [15] iteratively applies “expansion” and “inflation” steps to the transition matrix that denote the Markov chain of random walk. Reference [16] proposes a new spectral method based on the two-hop transition matrix of Markov random walk (SLCP2). In general, although much progress has been made, identifying protein complexes from PPI network still remains a challenge. The complexes derived by existing methods match few known complexes. The reason is that the topological structures of complexes are too complicated. It is difficult to define the topology by a specific type of pattern. It is necessary to develop a new method to avoid the problem of topological dependence.

In this paper, we present an optimization framework that uses a penalized least squares method to identify complexes from PPI network, named PLSMC. Intuitively, our method is on the basis of the fact that if two proteins are in a common complex, they are likely to be interacting [1, 4, 5]. PLSMC employs this relation to detect complexes using a penalized least squares method. By optimization, the propensities of proteins to complexes can be determined. The PLSMC is tested and compared with other methods on several public PPI networks of yeast. The results show that PLSMC has higher accuracy on matching with known complexes than other state-of-the-art methods. Moreover, the analysis of functional homogeneity indicates that complexes identified by PLSMC are biological relevance.

## 2. Materials and Methods

### 2.1. Penalized Least Squares Method for Complex Detection

In order to introduce our method, we first introduce several notations. A PPI network is denoted by a matrix of *G*_*N*×*N*_, where *N* is the number of proteins and *G*_*ij*_ is equal to 1 if proteins *i* and *j* are interacting, 0 otherwise. Since an interaction may be a false positive one when the corresponding proteins share less common interacting partners, we compute the weight matrix *S* for a PPI network as in [17],

(1) $$\begin{array}{c} S_{ij}=\{\begin{array}{cc} \frac{\left|N\left(i\right)\cap N\left(j\right)\right|}{\sqrt{\left|N\left(i\right)\right|\times \left|N\left(j\right)\right|}}, & \text{if}\;G_{ij}=1,\\ 0, & \text{otherwise}, \end{array} \end{array}$$

where *N*(*i*) is a set consisting of protein *i* and all of its neighbors.

Let *θ*_*iz*_ (*θ*_*iz*_ > 0) be the propensity denoting how likely protein *i* belongs to complex *z*, which is an unknown variable needing to be estimated. The cocomplex coefficient *C*_*ij*_ of proteins *i* and *j* denotes the likelihood that they participate in the same complexes. Given that there are at most *K* complexes existing in the PPI network, *C*_*ij*_ is calculated as

(2) $$\begin{array}{c} C_{ij}=\overset{K}{\underset{z=1}{\sum }}\theta_{iz}\theta_{jz}. \end{array}$$

Hence, the sum of distances between interaction weights and cocomplex coefficients over all pairs of proteins can be written as follows:

(3) $$\begin{array}{c} L=\overset{N}{\underset{i,j}{\sum }}\frac{1}{2}{\left(C_{ij}-S_{ij}\right)}^{2}=\overset{N}{\underset{i,j}{\sum }}\frac{1}{2}{\left(\overset{K}{\underset{z=1}{\sum }}\theta_{iz}\theta_{jz}-S_{ij}\right)}^{2}. \end{array}$$

Minimizing *L* with respect to Θ = [*θ*_*iz*_] is to make the cocomplex coefficient close to interaction weight for each pair of proteins. If two proteins are not interacting, the cocomplex coefficient of them is supposed to be minimized to 0. However, only considering the cocomplex coefficient is not sufficient for complex detection, since a protein may have large number of propensities with high values. It will assign a protein to too many complexes and thus produce pervasive overlapping complexes. Therefore, to control overlapping rate, we augment (3) with a penalty term to shrink the propensities as in (4). Consider

(4) $$\begin{array}{c} L=\overset{N}{\underset{i,j}{\sum }}\frac{1}{2}{\left(\overset{K}{\underset{z}{\sum }}\theta_{iz}\theta_{jz}-S_{ij}\right)}^{2}+\lambda \overset{N}{\underset{i}{\sum }}\overset{K}{\underset{z}{\sum }}\theta_{iz}^{2}, \end{array}$$

where *λ* (*λ* > 0) is the parameter of the penalization. Finally, the optimization in PLSMC is written as

(5) min⁡Θ L(Θ)=∑i,jN12(∑zKθizθjz−Sij)2+λ∑iN∑zKθiz2,s.t.    Θ≥0.

### 2.2. Estimating Protein Propensities

Estimating the propensities Θ = [*θ*_*iz*_] in (5) is a nonnegative constrained optimization problem. Let Φ = [*ϕ*_*iz*_] be the Lagrange multiplier for the constraint Θ ≥ 0. The Lagrange function *L* is as

(6) L(Θ,Φ)=∑i,jN12(∑zKθizθjz−Sij)2+λ∑iN∑zKθiz2 +∑iN∑zKϕizθiz.

Taking the derivation of (6) with respect to *θ*_*ik*_ and setting it to zero give

(7) $$\begin{array}{c} 2\overset{N}{\underset{j}{\sum }}\theta_{jk}\overset{K}{\underset{z}{\sum }}\theta_{iz}\theta_{jz}-2\overset{N}{\underset{j}{\sum }}\theta_{jk}S_{ij}+2\lambda \theta_{ik}+\phi_{ik}=0. \end{array}$$

It is difficult to estimate *θ*_*ik*_ in above equation using an analytical method, as it depends on *θ*_*jz*_, where *j* ≠ *i* and *z* ≠ *k*. Therefore, we use an iterative method, to find the optimal *θ*_*ik*_. Because *θ*_*ik*_ *ϕ*_*ik*_ = 0 for the Karush-Kuhn-Tucker condition, we multiply both sides of the equation by *θ*_*ik*_ and get

(8) $$\begin{array}{c} \theta_{ik}\left(\overset{N}{\underset{j}{\sum }}\theta_{jk}\overset{K}{\underset{z}{\sum }}\theta_{iz}\theta_{jz}+\lambda \theta_{ik}\right)=\theta_{ik}\overset{N}{\underset{j}{\sum }}\theta_{jk}S_{ij}. \end{array}$$

Then, we can write the multiplicative updating rule as

(9) $$\begin{array}{c} \theta_{ik}^{\text{new}}⟵\theta_{ik}\frac{\sum_{j}^{N}\theta_{jk}S_{ij}}{\sum_{j}^{N}\theta_{jk}\sum_{Z}^{K}\theta_{iz}\theta_{jz}+\lambda \theta_{ik}}. \end{array}$$

As suggested in the literature [18], we use the updating rule as

(10) $$\begin{array}{c} \theta_{ik}^{\text{new}}⟵\frac{\theta_{ik}}{2}+\frac{\theta_{ik}}{2}\frac{\sum_{j}^{N}\theta_{jk}S_{ij}}{\sum_{j}^{N}\theta_{jk}\sum_{Z}^{K}\theta_{iz}\theta_{jz}+\lambda \theta_{ik}}. \end{array}$$

With the updating rule, we could estimate the propensities *θ*_*ik*_. The reason why we use the multiplicative updating rule is that it is a gradient descent method with an adaptive step length and is guaranteed to converge to an optimum [19–21].

### 2.3. Postprocessing

After estimating the propensities, we could obtain complexes using the estimated propensity matrix Θ = [*θ*_*ik*_]. We introduce a propensity threshold *τ* to derive the complexes. If *θ*_*ik*_ ≥ *τ*, the protein *i* is allocated to the complex *k*. Thus, a set of predicted complexes *C* in the network *G* is obtained, in which each element consists of a group of proteins. Moreover, as previous methods, the predicted complexes in set *C* that include less than 3 proteins are removed.

### 2.4. A Speeding-Up Strategy

The time-consuming is prohibitive when the optimizing process is directly conducted on a large-scale real world PPI network. Therefore, it is appropriate to execute the estimating process on a set of subnetworks that are of small scale but enough to identify complexes. To get the subnetworks, we recursively cluster the network into subnetworks containing proteins less than a specific size *N*_*s*_. Then, apply the optimization procedure to each subnetwork to detect complexes. We use the tool of fastCommunity [22] to cluster the network. The reason is that it is a fast and robust algorithm in the field of network clustering.

In particular, we first use fastCommunity to cluster the input network and let each cluster be a subnetwork. Redo the process on each subnetwork larger than *N*_*s*_, until there is no subnetwork larger than *N*_*s*_.

### 2.5. PLSMC Algorithm

Three main steps in PLSMC are as follows: (1) get subnetworks from the input PPI network; (2) compute the weight matrix and initialize the propensity matrix with random values for each subnetwork; (3) estimate protein propensities in each subnetwork; (4) identify complexes of proteins using the postprocessing step. The pseudocode of PLSMC is in Algorithm 1.

## 3. Results and Discussion

We implemented a Java archive and a Web tool of the PLSMC algorithm, which is available at http://nclab.hit.edu.cn/PLSMC/. To examine its effectiveness, PLSMC is tested on several public PPI networks of yeast and compared with some state-of-the-art methods. The matching with known complexes and functional homogeneity of predicted complexes are both studied.

### 3.1. Dataset and Evaluation Metrics

We investigate the performance on several PPI networks of yeast (*Saccharomyces cerevisiae*), including Krogan [23], Collins [24], Gavin [2], and BioGRID [25] datasets. For Krogan, we use high confidence interactions with the probability higher than 0.273. For Gavin, only interactions with socioaffinity index larger than 5 are considered. For Collins network, we choose the top 9074 interactions with respect to purification enrichment score. The above cutoffs are suggested by original papers and [14]. In addition, all of physical interactions in BioGRID dataset (version 3.1.92) are downloaded. The general characteristics of these networks are listed in Supplementary Table S1 available online at http://dx.doi.org/10.1155/2014/720960.

The matching between predicted complexes and known complexes is studied to evaluate the accuracy of the prediction. We use CYC2008 catalogue [26] as the gold standard of known complexes in this work, which is available at http://wodaklab.org/cyc2008/. The CYC2008 includes the complexes that are all validated by small-scale experiments and it is an up-to-date comprehensive dataset of known complexes of yeast. As in the literature [14], the known complexes in CYC2008 containing less than 3 proteins are removed.

Three metrics in the following are used to evaluate the accuracy of matching between a predicted complex set *P* and a gold standard *B*.

#### 3.1.1. *f*-Measure

A predicted complex *p* ∈ *P* and a known one *b* ∈ *B* are considered to be matching, if the overlapping score* os *(*p*, *b*) is greater than a matching threshold* ov* (*ov* is set to 0.25 as in [4]). The overlapping score is defined as

(11) $$\begin{array}{c} os\left(p,b\right)=\frac{{\left|p\cap b\right|}^{2}}{\left(\left|p\right|\times \left|b\right|\right)}. \end{array}$$

Let *N*_*cp*_ be the number of predicted complexes that match at least one known complex and let *N*_*cb*_ be the number of known complexes that match at least one predicted complex. The precision and recall are defined as follows:

(12) $$\begin{array}{c} \text{precison}=\frac{N_{cp}}{\left|P\right|},\;\;\text{recall}=\frac{N_{cb}}{\left|B\right|}. \end{array}$$

The *f*-measure is the harmonic mean of precision and recall as

(13) $$\begin{array}{c} f\text{-measure}=\frac{2\times \text{precision}\times \text{recall}}{\left(\text{precision}+\text{recall}\right)}. \end{array}$$

#### 3.1.2. Acc Metric

Let *T*_*ij*_ be the number of common proteins between a known complex *i* and a predicted complex *j*. Then, the sensitivity (Sn) and positive predictive value (PPV) are as follows:

(14) $$\begin{array}{c} \text{Sn}=\frac{\left(\sum_{i=1}^{\left|B\right|}\max_{j}\left\{T_{ij}\right\}\right)}{\left(\sum_{i=1}^{\left|B\right|}N_{i}\right)},\\ \text{PPV}=\frac{\left(\sum_{j=1}^{\left|P\right|}\max_{i}\left\{T_{ij}\right\}\right)}{\left(\sum_{i=1}^{\left|B\right|}\sum_{j=1}^{\left|P\right|}T_{ij}\right)}, \end{array}$$

where *N*_*i*_ is the number of proteins in a known complex *i*. Then, the accuracy metric [14] is defined as

(15) $$\begin{array}{c} \text{Acc}=\sqrt{\text{Sn}\times \text{PPV}}. \end{array}$$

#### 3.1.3. MMR Metric

Recently, [14] proposed a novel metric called maximum matching ratio (MMR) as follows:

(16) $$\begin{array}{c} \text{MMR}=\frac{\sum_{i=1}^{\left|B\right|}\max_{j=1}^{\left|P\right|}os\left(p_{j},b_{i}\right)}{\left|B\right|}, \end{array}$$

where *b*_*i*_ and *p*_*j*_ are *i*th known complex in *B* and *j*th predicted complex in *P*, respectively.

It is important to note that each of above evaluation metrics does not provide an adequate description of the matching between predicted complexes and known complexes. To make a comprehensive evaluation, we consider the composite score that is the sum of above three scores in this study. Similar composite score is also used in the literature [14].

### 3.2. Investigation of PLSMC

The parameter *N*_*s*_ in PLSMC controls the size of subnetwork and is significantly related to the effect of the speed-up strategy. We test different values of *N*_*s*_ = {50,100,200,300,400,500}. Because of the prohibitive cost of computation, *N*_*s*_ larger than 500 is not investigated. For each value of *N*_*s*_, we try different values of penalty parameter *λ* (*λ* ∈ {2^−5^,…, 2^5^}) and repeat executing the algorithm 100 times with random initialization. We choose the execution that the estimated propensity matrix gives the minimal value of *L* in (5). We choose the values of propensity threshold *τ* from 0.05 to 0.5 with increment 0.05 that gives the best composite score. Supplementary Table S2 shows the best parameter setting for each value of *N*_*s*_.

**Figure 1:**
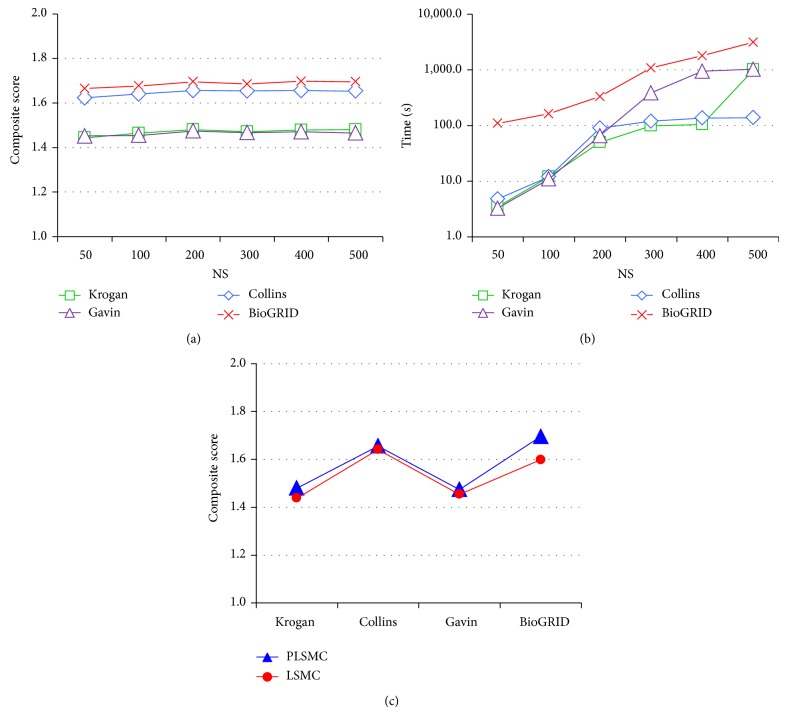
Comparison of PLSMC with different parameter setting. (a) and (b) are the comparison of composite score and execution time of PLSMC with different value of *N*_*s*_ (max size of subnetwork) applied to the four networks. (c) is the composite scores of PLSMC and PLSMC without the penalty term (denoted by LSMC).

We demonstrate the effect of *N*_*s*_ with different values on the four networks in Figures 1(a) and 1(b). As in Figure 1(a), on all networks, the composite score decreases with the parameter *N*_*s*_ when *N*_*s*_ ≤ 200 and fluctuates when *N*_*s*_ > 200. Meanwhile, the execution time increases with the parameter dramatically as in Figure 1(b). It indicates that the speed-up procedure could make a good balance between the computation time and prediction performance when *N*_*s*_ = 200. Interestingly, this is also consistent with that in CYC2008 [26], in which there is no known complex including more than 200 proteins. Therefore, in the following of this study, *N*_*s*_ is set to 200.

To examine the effect of the penalty term introduced in (4), we compare the PLSMC using the term and the one without using it (denoted by LSMC) applied to the four networks. The parameter setting of LSMC is shown in Supplementary Table S3.  Figure 1(c) illustrates the results of PLSMC and LSMC. As shown, the PLSMC outperforms LSMC applied to all four networks. This confirms that the penalty term in (4) is essential.

### 3.3. Comparison with Other Methods on Matching Known Complexes

We compare PLSMC with SLCP2 [16], ClusterONE [14], RSGNM [21], OCG [12], MCL [15], and CFinder [10]. The parameters of these algorithms are tuned as follows: ClusterONE: density (*d*) and merging threshold (*mo*) both from 0.1 to 1.0 with increment 0.1; RSGNM: rate parameter *β* ∈ {2^−5^,…, 2^5^} and the parameter *λ* ∈ {2^−5^,…, 2^5^}; MCL: inflation from 1.2 to 5.0 with increment of 0.1; CFinder: the size (*k*) of *k*-clique is changed from 3 to 10; OCG: using centered cliques initialization and modularity maximization; SLCP2: no parameter needs to be tuned. We remove the predicted complexes of above methods with size smaller than 3 and choose the parameter setting that yields the best composite score. The general information including parameter settings of the algorithms applied to four networks is in Supplementary Table S4, where (Com.) is the number of predicted complexes, (Prot.) is the number of covered proteins, and (Size) is the average size of predicted complexes. We cannot obtain the results of CFinder on BioGRID network, as the calculation requires more memory than a typical computer.

**Figure 2:**
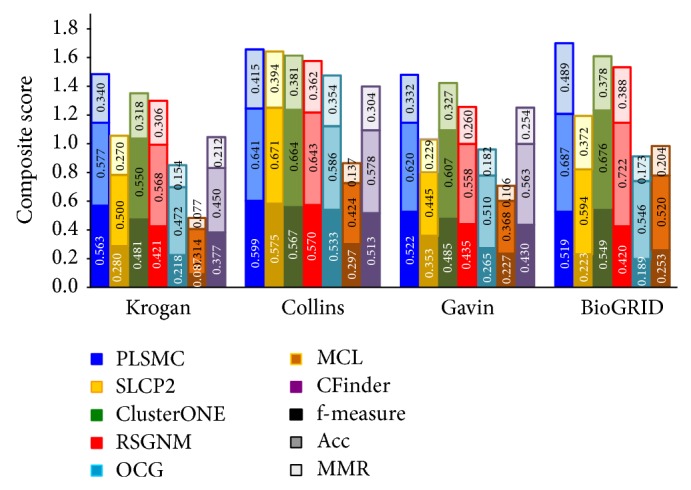
Comparison on composite score of the algorithms applied to four networks. Various shades of the same color denote *f*-measure, Acc, and MMR submetrics. The total height of each bar is the value of composite score.

We present the comparison result of matching with gold standard in Figure 2. On all four networks, PLSMC could get better composite score than other methods. ClusterONE gets close results to PLSMC on all networks. SLCP2 and OCG provide good performance when applied to Collins network but make poor predictions about other networks. It indicates that these two methods are prone to be affected by different networks. MCL achieves poor performance when applied to all networks.

**Figure 3:**
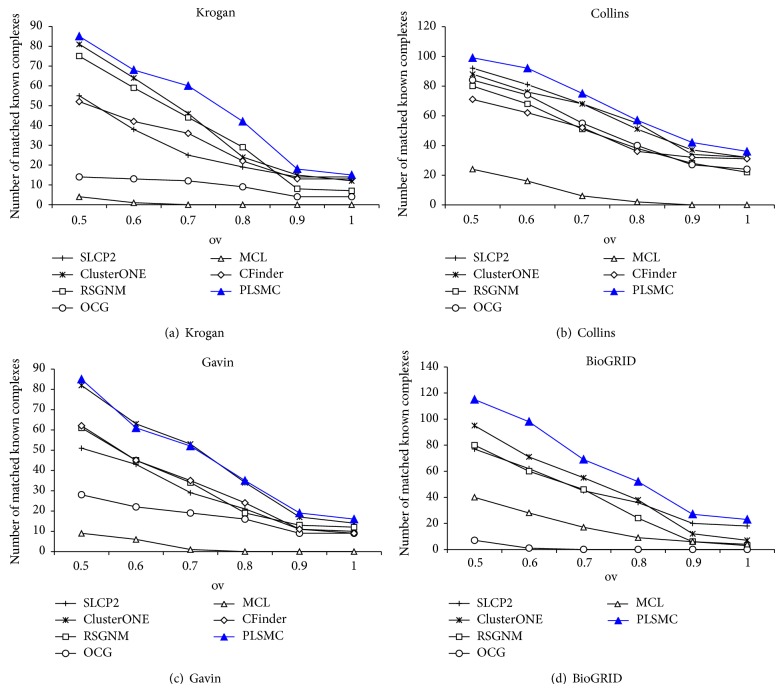
The number of matched known complexes of the algorithms.

In addition, we also investigate the number of known complexes that are matched by predicted complexes. The number of matched known complexes of various algorithms applied to Krogan, Collins, Gavin, and BioGRID networks is illustrated in Figures 3(a)–3(d), respectively. We show the results of the overlapping threshold *ov* from 0.5 to 1.0. It denotes a perfect matching when *ov* = 1. As shown, PLSMC can hit 15, 36, 16, and 23 known complexes with perfect matching on four networks, respectively. It can also be found that, on Krogan, Collins, and BioGRID networks, PLSMC can provide the greatest number using all thresholds. On Gavin network, PLSMC could get comparative results with ClusterONE with all thresholds and match more known complexes with perfect matching than others. Generally, the above comparisons confirm that the PLSMC outperforms other methods in terms of matching known complexes in gold standard.

**Figure 4:**
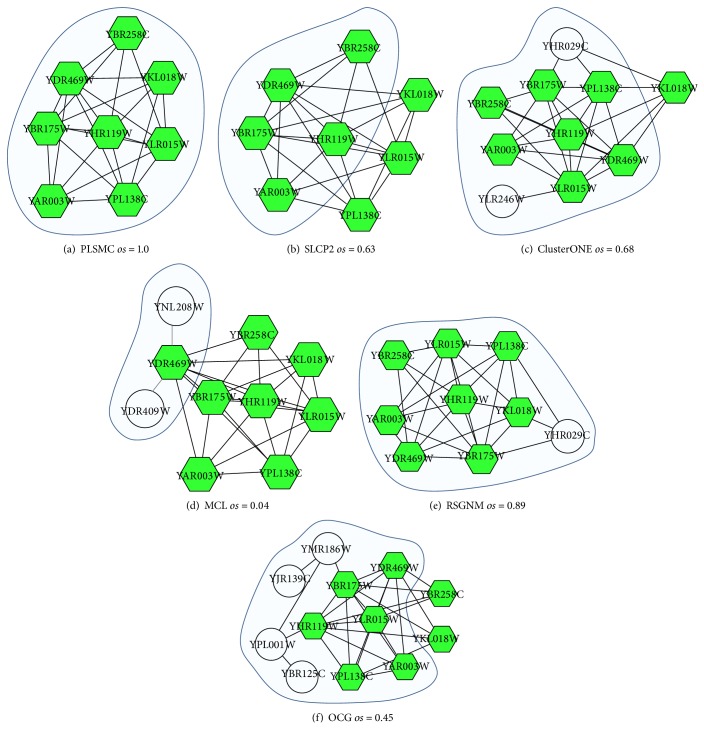
The COMPASS complex as detected by the six algorithms. Hexagon nodes represent the proteins involved in the COMPASS complex. Shaded areas are the clusters detected by the algorithms, which have the max overlapping scores (*os*) with COMPASS complex.

We show how the studied algorithms identify the known COMPASS complex from the Krogan network in Figure 4. The COMPASS complex is an important conserved protein complex that catalyzes methylation of histone H3, which is collected in both CYC2008 and GO (GO: 0048188). The complex contains 8 proteins (YKL018W, YPL138C, YBR175W, YDR469W, YHR119W, YLR015W, YAR003W, and YBR258C), which are denoted by hexagon nodes in Figure 4. The clusters under the shaded areas are detected by the algorithms, which have the max overlapping scores (*os*) with COMPASS complex. As shown, PLSMC is the only algorithm that is able to detect this complex with perfect matching. All of the other algorithms make inaccurate prediction. SLCP2 detects a part of the complex and other algorithms include unrelated proteins into the complex. The result of CFinder is not shown, because the detected cluster that has the best matching with the complex is a huge cluster, which consists of 627 proteins.

### 3.4. Biological Relevance of Predicted Complexes

The known complex dataset is incomplete. For example, CYC2008 only covers 1627 proteins, while the number of proteins in yeast is more than 5000. Therefore, a predicted complex that does not match with any known complex is possibly not a false positive one and it is worth further in-depth analysis. To this end, we also examine the biological relevance of predicted complexes in terms of functional homogeneity. This is because the proteins within a complex tend to be located in the same cellular component (CC) or are involved in a common molecular function (MF) or biological process (BP) [4, 14]. We use the tool of GO::TermFinder (Version 0.83) [27] to compute the *P* value for each predicted complex. The GO corpus is downloaded from Saccharomyces Genome Database [28]. We investigate all three aspects of GO.

A predicted complex that has more than one annotation with the *P* value smaller than a threshold *p* is considered functional homogeneity. The threshold *p* is set to 1.0*E* − 10 [4]. The fraction of predicted complexes that are functional homogeneity is used to evaluate the performance of the prediction method.

Table 1 presents the comparison of functional homogeneity of complexes predicted by different methods. The result of known complexes in CYC2008 is also listed. It can be found that the complexes predicted by PLSMC are more functional homologous than those of other methods. Moreover, the results of PLSMC applied to Krogan, Collins, and Biological networks are all better than that of CYC2008. More interestingly, on all networks, the results of PLSMC in regard to CC aspect are better than MF and BP aspects. This tendency is consistent with that of CYC2008. On the whole, the comparison demonstrates that the complexes derived by PLSMC are more biologically relevant.

## 4. Conclusion

In this paper, we present PLSMC, a penalized least squares method, to detect complexes from PPI network. PLSMC identifies complexes by minimizing the distances between cocomplex coefficients and interaction weights of all pairs of proteins. We test it on several yeast PPI networks. The results show that PLSMC achieves higher accuracy in matching with known complexes than some state-of-the-art methods. Moreover, the predicted complexes also have good biological relevance to functional homogeneity. This study confirms that PLSMC, based on a least squares method, is an effective approach to identify complexes from the PPI network.

We note that integrating multiple biological data sources in addition to PPI network [29] can improve the identification of protein complexes. On the one hand, most of available protein-protein interaction networks are static. Combining dynamic information such as expression profiles can infer the dynamic properties of protein-protein interactions under different time points or various conditions [1, 30]. On the other hand, when two or more proteins form a complex, some interface information as physical folds [31], biochemical properties [32], and posttranslation modifications [33] is very important to the complex formation. In the future, based on PLSMC, we will study the identification of protein complexes from dynamic protein-protein interaction networks and interface datasets.

## Supplementary Material

Table S1. Is the basic properties of PPI networks used in this study.
Table S2. Shows the parameter settings of PLSMC with different values of Ns applied to four networks.
Table S3. Presents the parameter settings of LSMC applied to four networks.
Table S4. Is the general characteristics and parameter settings of PLSMC as well as other algorithms applied to four networks.

## Figures and Tables

**Algorithm 1:**
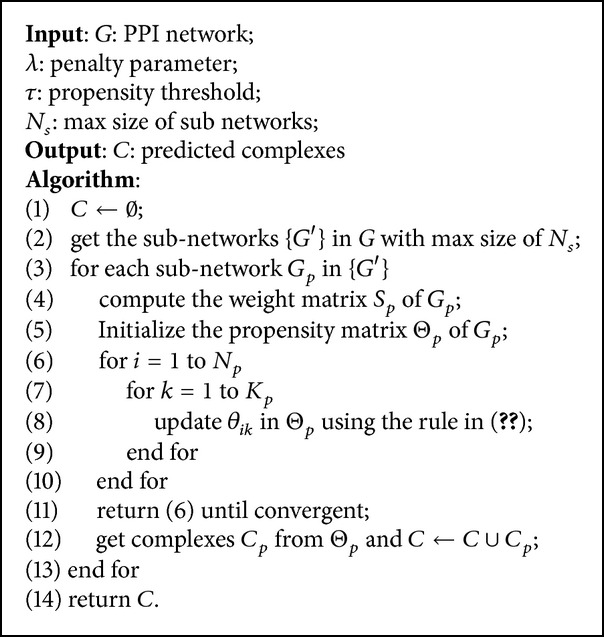
PLSMC (*G*, *λ*, *τ*, *N*_*s*_).

**Table 1:** Comparison on biological relevance of complexes predicted by the algorithms.

Network	Method	MF	BP	CC
Krogan	**PLSMC**	**0.479 **	**0.457 **	**0.592 **
	SLCP2	0.394	0.114	0.094
	ClusterONE	0.311	0.291	0.357
	RSGNM	0.392	0.270	0.270
	OCG	0.199	0.185	0.331
	MCL	0.265	0.057	0.033
	CFinder	0.296	0.287	0.330

Collins	**PLSMC**	**0.536 **	**0.460 **	**0.620 **
	SLCP2	0.405	0.353	0.410
	ClusterONE	0.401	0.377	0.419
	RSGNM	0.376	0.371	0.418
	OCG	0.519	0.439	0.612
	MCL	0.380	0.240	0.331
	CFinder	0.439	0.351	0.439

Gavin	**PLSMC**	**0.399 **	**0.362 **	**0.467 **
	SLCP2	0.374	0.153	0.189
	ClusterONE	0.374	0.308	0.360
	RSGNM	0.382	0.333	0.389
	OCG	0.381	0.310	0.405
	MCL	0.308	0.112	0.210
	CFinder	0.387	0.350	0.401

BioGRID	**PLSMC**	**0.459 **	**0.452 **	**0.511 **
	SLCP2	0.443	0.184	0.117
	ClusterONE	0.439	0.447	0.439
	RSGNM	0.363	0.277	0.267
	OCG	0.262	0.321	0.343
	MCL	0.400	0.176	0.140
	CFinder	—	—	—

CYC2008		0.458	0.424	0.525

MF, molecular function; BP, biological process; CC, cellular compartment.
