# Supplementary material for: A Least Square Method Based Model for Identifying Protein Complexes in Protein-Protein Interaction Network
Source: Biomed Res Int. 2014 Oct 23;2014:720960. doi: 10.1155/2014/720960 (PMC4227386; doi:10.1155/2014/720960)
Supplement: Supplementary file 1 — Table S1. Is the basic properties of PPI networks used in this study. Table S2. Shows the parameter settings of PLSMC with different values of Ns applied to four networks. Table S3. Presents the parameter settings of LSMC applied to four networks. Table S4. Is the general characteristics and parameter settings of PLSMC as well as other algorithms applied to four networks. [file 720960.f1.doc]

**Supplementary Table S1. Detail information of PPI networks.**

| Network | Proteins | Interactions |
| --- | --- | --- |
| Krogan | 2708 | 7123 |
| Collins | 1622 | 9074 |
| Gavin | 1855 | 7669 |
| BioGRID | 5640 | 59748 |

**Supplementary Table S2.** Parameter settings of PLSMC with various values of *N_s_* applied to four networks.

| Network | *N_s_* | Parameter Setting |
| --- | --- | --- |
|  |  |  |
| Krogan | 50 | *λ* = 0.5 *τ* = 0.15 |
|  | 100 | *λ* = 1.0 τ = 0.1 |
|  | **200** | ***λ* = 1.0 τ = 0.1** |
|  | 300 | *λ* = 1.0 τ = 0.1 |
|  | 400 | *λ* = 0.5 τ = 0.1 |
|  | 500 | *λ* = 1.0 τ = 0.1 |
| Collins | 50 | *λ* = 1.0 τ = 0.25 |
|  | 100 | *λ* = 0.0625 τ = 0.1 |
|  | **200** | ***λ* = 0.0625 τ = 0.1** |
|  | 300 | *λ* = 0.0625 τ = 0.05 |
|  | 400 | *λ* = 0.0625 τ = 0.15 |
|  | 500 | *λ* = 0.0625 τ = 0.05 |
| Gavin | 50 | *λ* = 1.0 τ = 0.2 |
|  | 100 | *λ* = 0.5 τ = 0.15 |
|  | **200** | ***λ =* 1.0 τ = 0.2** |
|  | 300 | *λ =* 1.0 τ = 0.25 |
|  | 400 | *λ =* 1.0 τ = 0.15 |
|  | 500 | *λ =* 1.0 τ = 0.15 |
| BioGRID | 50 | *λ* = 1.0 τ = 0.2 |
|  | 100 | *λ* = 1.0 τ = 0.15 |
|  | **200** | ***λ* = 0.5 τ = 0.25** |
|  | 300 | *λ* = 1.0 τ = 0.2 |
|  | 400 | *λ* = 1.0 τ = 0.2 |
|  | 500 | *λ* = 1.0 τ = 0.2 |

**Supplementary Table S3. Parameter settings of LSMC applied to four networks.**

| Network | Parameter Setting |
| --- | --- |
| Krogan | *τ* = 0.1 |
| Collins | *τ* = 0.05 |
| Gavin | *τ* = 0.15 |
| BioGRID | *τ* = 0.2 |

**Supplementary Table S4. The general characteristics and parameter settings of the algorithms applied to four networks.**

| Network | Method | Parameter | Com. | Prot. | Size |
| --- | --- | --- | --- | --- | --- |
| Krogan | PLSMC | *λ =* 1.0, *τ =* 0.1 | 422 | 1046 | 7.9 |
|  | SLCP2 | *--* | 437 | 1683 | 3.9 |
|  | ClusterONE | *mo*=0.9 *d*=0.6 | 244 | 1074 | 5.3 |
|  | RSGNM | *β*=2  *λ*=16 | 237 | 1213 | 5.6 |
|  | OCG | *Cen. Mod.* | 302 | 2436 | 16.3 |
|  | MCL | *Inflation*=1.5 | 211 | 856 | 4.2 |
|  | CFinder | *k* = 3 | 115 | 1143 | 10.9 |
| Collins | PLSMC | *λ=*0.0625, *τ=*0.1 | 237 | 1037 | 8.7 |
|  | SLCP2 | *--* | 173 | 1368 | 7.9 |
|  | ClusterONE | *mo*=0.8 *d*=0.2 | 167 | 1350 | 8.9 |
|  | RSGNM | *β*=4 *λ*=4 | 170 | 1264 | 8.6 |
|  | OCG | *Cen. Mod.* | 312 | 1260 | 13.1 |
|  | MCL | *Inflation*=1.5 | 121 | 804 | 6.8 |
|  | CFinder | *k* = 3 | 114 | 1161 | 10.6 |
| Gavin | PLSMC | *λ =* 1.0, *τ =* 0.2 | 323 | 1204 | 8.4 |
|  | SLCP2 | *--* | 190 | 734 | 3.9 |
|  | ClusterONE | *mo*=0.9 *d*=0.8 | 214 | 1186 | 7.0 |
|  | RSGNM | *β*=16 *λ*=4 | 144 | 999 | 7.4 |
|  | OCG | *Cen. Mod.* | 210 | 1707 | 16.3 |
|  | MCL | *Inflation*=1.7 | 143 | 683 | 4.8 |
|  | CFinder | *k* = 4 | 137 | 1158 | 9.6 |
| BioGrid | PLSMC | *λ =* 0.5, *τ =* 0.25 | 595 | 1847 | 8.5 |
|  | SLCP2 | *--* | 810 | 3721 | 4.6 |
|  | ClusterONE | *mo*=0.9 *d*=0.8 | 246 | 1580 | 8.5 |
|  | RSGNM | *β*=4 *λ*=4 | 405 | 2924 | 7.8 |
|  | OCG | *Cen. Mod.* | 580 | 5617 | 23.6 |
|  | MCL | *Inflation*=3.2 | 335 | 3652 | 10.9 |

Com. is the number of predicted complexes. Prot. is the number of covered proteins. Size is the average size of predicted complexes.
